# Snoring during pregnancy and its relation to sleepiness and pregnancy outcome - a prospective study

**DOI:** 10.1186/1471-2393-14-15

**Published:** 2014-01-13

**Authors:** Maria Sarberg, Eva Svanborg, Ann-Britt Wiréhn, Ann Josefsson

**Affiliations:** 1 Division of Obstetrics and Gynecology, Department of Clinical and Experimental Medicine, Faculty of Health Sciences, Linköping University, Department of Obstetrics and Gynecology in Linköping, County Council of Östergötland, Linköping, Sweden; 2 Department of Clinical Neurophysiology, Faculty of Health Sciences, Linköping University, County Council of Östergötland, Linköping, Sweden; 3 Local Health Care Research and Development Unit, Faculty of Health Sciences, Linköping University, County Council of Östergötland, Linköping, Sweden

**Keywords:** Pregnancy, Snoring, Sleepiness, Epworth sleepiness score, Body mass index, Edema, Pregnancy outcome

## Abstract

**Background:**

The incidence of snoring and sleepiness is known to increase during pregnancy, and this might impact maternal health and obstetric outcome. However, the association between snoring and sleepiness during pregnancy is not fully understood. This study was aimed at investigating the development of snoring during pregnancy and prospectively assessing if there is an association between snoring and sleepiness or adverse pregnancy outcomes, such as preeclampsia, mode of delivery, and fetal complications.

**Methods:**

Consecutively recruited pregnant women (n = 500) received a questionnaire concerning snoring and sleep at the 1^st^ and 3rd trimester of pregnancy. The women who had rated their frequency of snoring at both occasions (n = 340) were divided into subgroups according to the development of snoring they reported and included in the subsequent analyses. Additional medical data were collected from the medical records.

**Results:**

The frequency of snoring was 7.9% in the 1^st^ trimester and increased to 21.2% in the 3^rd^ trimester of pregnancy. The women who snored already in early pregnancy had significantly higher baseline BMI (p = 0.001) than the women who never snored, but snoring was not associated with the magnitude of weight gain during pregnancy. Snoring women were more likely to experience edema in late pregnancy than the non-snorers. Women who started to snore during pregnancy had higher Epworth Sleepiness Scores than the non snorers in both early and late pregnancy. No significant association between obstetric outcome and snoring was found.

**Conclusion:**

Snoring does increase during pregnancy, and this increase is associated with sleepiness, higher BMI at the start of pregnancy and higher prevalence of edema, but not with weight gain.

## Background

Sleep problems are common during pregnancy, characterized by declining sleep quality, increased night wakening and snoring [1-3]. A prospective American study from 1996 reported that snoring increased from 4% before pregnancy to 14% in the last trimester of pregnancy [4]. Later prospective studies report snoring rates increasing from 3.7% [5] and 7.1% [6] to 11.8% and 13% respectively. Results from cross sectional studies conducted in late pregnancy describe an incidence of habitual snoring from 11.9% to 49% [7-10] in 3^rd^ trimester of pregnancy, and retrospective studies, where the women were recruited after delivery, report a range of 2.5-35.1% snorers in late pregnancy [11-14].

As a result of snoring and other sleep related problems during pregnancy daytime sleepiness and fatigue may occur [15], especially when associated with obstructive sleep apnea (OSAS) [16]. The sleepiness is mainly due to fragmented sleep caused by repeated arousals. It is well known that women are getting more sleepy and tired when pregnant [3], but studies on a possible relation between snoring and sleepiness during pregnancy are few and retrospective [17,18]. Sleep deprivation and sleep disturbances during pregnancy may increase the risk of adverse pregnancy outcomes e.g. gestational hypertension, preeclampsia, gestational diabetes, preterm delivery, unplanned cesarean deliveries, growth restriction of the fetus and postpartum depression [10,14,19-24]. Various explanations for potential mechanisms by which sleep related problems might exert a negative effect on outcome have been proposed. However, direct evidence is scarce [21-23]. This study was conducted to prospectively examine the possible relation between snoring and sleepiness and adverse obstetric outcomes over the entire pregnancy, assessed in the same women in the 1^st^ and 3^rd^ trimester as well as postpartum.

Hence, the primary aim was to determine the frequency and development of snoring during pregnancy. The secondary aim was to investigate if associations between snoring, sleepiness and an adverse pregnancy outcome could be verified in a prospective study.

## Methods

Pregnant women consecutively registered at one antenatal care clinic (ACC) during 2007 were asked to contribute to the study. The Swedish antenatal health care system reaches almost 100% of all pregnant women and is free of charge. At the ACC healthy pregnant women are advised to attend the regular antenatal program consisting of seven to nine visits to a midwife, and, if needed, to make extra appointments with an obstetrician and/or the midwife [25]. The women were asked to join the study at their first visit to the ACC, which usually takes place around gestational week 10 –12. Women with diabetes mellitus, neurological disease, drug abuse, hypertension or poor knowledge of the Swedish language at the first visit were excluded. After receiving written and oral information 500 women agreed to participate in the study. Written informed consent was obtained from each participant.

The contributing women were given a questionnaire at two regular visits to the ACC, in the 1^st^ and 3^rd^ trimester respectively. In the questionnaire they were asked to rate the frequency with which they experienced snoring on a daily bases, rated in terms of “always”, “often”, “sometimes”, “seldom” and “never”. The degree of edema experienced in the feet, legs or hands was rated as “none”, “mild”, “moderate” or “severe” edema. The questionnaire also contained the Epworth Sleepiness Scale, a standardized instrument for measuring sleepiness among patients with snoring and obstructive sleep apnea [26]. In the Epworth Sleepiness Scale the women rank their probability of falling asleep on a scale of increasing probability from 0 to 3 for eight different situations. Maximum score is 24; a score ≥10 is considered abnormal and defined as excessive daytime sleepiness (EDS). Furthermore, the women were asked the four separate questions set by the International RLS Study Group for diagnosing restless legs syndrome [27].

The women’s body weight and blood pressure were recorded at all visits where questionnaires were distributed. Data concerning the characteristics of the women and their pregnancies (age, marital status, height, parity, existence of twin pregnancies, gestational weeks at delivery, mode of delivery, diagnosed complications of pregnancy) and their newborn (sex, weight, Apgar score) were taken from the Swedish standardized antenatal and delivery records.

The participating women were divided into three subgroups depending on how their snoring had developed during pregnancy. Women who reported that they snored “never”, “seldom” or “sometimes” in both the 1^st^ and 3^rd^ trimester were identified as “non snorers” and women who snored “often” or “always” already in the 1^st^ trimester of pregnancy were identified as “habitual snorers”. Women who started to snore during pregnancy, i.e. snored “never”, “seldom” or “sometimes” in the first trimester and “often” or “always” in the 3^rd^ trimester were considered “gestational snorers”. Only the women who had answered the question about snoring in both questionnaires were included in these groups and further analyses.

Gestational hypertension was defined as systolic blood pressure ≥140 or diastolic blood pressure ≥ 90. Diagnosis of preeclampsia required additional leakage of protein to urine ≥0.3 gram/24 hours according to the ICD-10 criteria [28].

Characteristics of the pregnant women were presented as mean and standard deviation (SD) for continuous variables and as numbers and percentages for discrete variables. In comparisons between the three groups one-way analysis of variance (ANOVA) and Turkey’s range test for post hoc analysis were used for comparing mean values and binary variables. The χ^2^ test was used for comparison of categorized variables between the three groups. For analyzing changes in the entire material independent two sample t-test was used. The significance level was set to 5% in all tests. The statistical software SPSS 21.0 was used. The study was approved by The Regional Ethical Review Board, Linköping, Sweden (M97-05).

## Results

### Participants

In total 500 women answered the questionnaire in the 1^st^ trimester (mean gestational week 11.2, SD 1.6) and 351 women in the 3^rd^ trimester of pregnancy (mean gestational week 34.4, SD 0.6). One woman with an essential hypertension was included by mistake. Between enrollment and the third questionnaire, 11 women had a miscarriage or abortion, 11 had preterm labor, and 9 moved to another city. The causes of drop-out were unknown in the remaining cases. Eleven of the 351 women did not answer the question about snoring in one of the questionnaires. Therefore 340 women were included in further analyses. The drop-outs did not differ from the remaining women according to age (p = 0.85), BMI class at inclusion (p = 0.14), parity (p = 0.61) or prevalence of habitual snoring (p = 0.70). The included women had characteristics similar to those of average Swedish pregnant women [25]; their mean age was 30.0 years (range 19–47 years) at the start of pregnancy. The background data for the participating women are shown in Table 1.

**Table 1:** **Baseline characteristics** (**n** = **340**)

	**Non snorers**		**Gestational**		**Habitual snorers**		
	**(n = 268)**		**snorers (n = 45)**		**(n = 27)**		
	**n**	**%**	**n**	**%**	**n**	**%**	**p value**
**Age (years, mean/SD)**	30.2/4.33		29.4/4.08		29.6/5.67		0.481^1^
**Relationsship status**							
Partnered	267	99.6	44	97.8	24	88.9	0.915^2^
Single	1	0.4	1	2.2	3	11.1	<0.001^3^
**Primipara**							0.001^4^
Yes	122	45.5	22	48.9	15	55.6	0.583^1^
No	146	54.5	23	51.1	12	44.4	
**BMI at start of pregnancy (kg/m**^**2**^**; mean/SD)**	23.6/3.8		24.4/3.3		26.4/5.0		0.381^2^
							0.001^3^
							0.098^4^
**BMI classes at start of pregnancy**							0.001^5^
<19	11	4.3	0	0.0	0	0.0	
19-24	183	71.8	27	60.0	9	33.3	
25-29	56	22.0	15	33.3	13	48.1	
≥30	15	5.9	3	6.7	5	18.5	
**Twin pregnancy**	1	0.4	0	0.0	2	7.4	

^1^One-way analysis of variance, ANOVA.

^2^Post hoc analysis (Tukey): non snorers vs. gestational snorers.

^3^Post hoc analysis (Tukey): non snorers vs. habitual snorers.

^4^Post hoc analysis (Tukey): gestational snorers vs. habitual snorers.

^5^Chi square test.

### Snoring

The women were divided into three groups regarding their development of snoring during pregnancy: “non snorers” who never snored during pregnancy (n = 268), “gestational snorers” who started to snore between the 1^st^ and 3^rd^ trimester of pregnancy (n = 45) and “habitual snorers” who snored already from the 1^st^ trimester of pregnancy (n = 27). This gives a proportion of habitual snoring (snoring often or always) of 7.9% (n = 27) in the 1^st^ and 21.2% (n = 72) in the 3^rd^ trimester of pregnancy.

### BMI, weight gain and edema

The mean BMI in the entire group was 24.0 kg/m^2^. The “habitual snorers” had significantly higher mean BMI than the “non snorers” at the start of pregnancy (p = 0.001) and a greater share of the “habitual snorers” were classified as obese (Table 1). However, there was no difference in weight gain during pregnancy between the groups (Table 2). Both the “gestational snorers” and the “habitual snorers” experienced significantly more edema than the “non snorers” in the 3^rd^ trimester of pregnancy (p = 0.001 and 0.002, Table 2).

**Table 2:** **Pregnancy data** (**n** = **340**)

	**Non snorers**		**Gestational snorers**		**Habitual snorers**		
	**(n = 268)**		**(n = 45)**		**(n = 27)**		
	**n**	**%**	**n**	**%**	**n**	**%**	**p value**
**Weight gain (kg, mean/SD)**	10.4/3.4		11.5/3.8		11.2/3.9		0.107^1^
**Systolic blood pressure (mmHg, mean/SD)**							
1^st^ trimester	110.3/12.1		111.0/10.3		111.9/10.6		0.779^1^
3^rd^ trimester	112.7/9.1		114.1/9.8		110.9/9.1		0.358^1^
**Diastolic blood pressure (mmHg, mean/SD)**							
1^st^ trimester	66.0/7.6		67.0/6.7		69.6/8.0		0.053^1^
3^rd^ trimester	67.2/7.0		65.9/8.2		68.3/7.3		0.353^1^
**Edema (moderate or severe)**							
1^st^ trimester	10	3.8	3	6.7	2	7.4	0.505^1^
3^rd^ trimester	58	21.7	21	46.7	14	51.9	0.001^2^
							0.002^3^
							0.876^4^
**Epworth sleepiness score (ESS, mean/SD)**							
1^st^ trimester	7.6/3.6		9.3/3.0		8.7/3.3		0.009^2^
							0.264^3^
							0.784^4^
3^rd^ trimester	8.4/3.8		10.6/3.4		9.4/4.8		0.001^2^
							0.388^3^
							0.382^4^
**Epworth sleepiness score** ≥**10 (EDS)**							
1^st^ trimester	81	30.2	17	37.8	9	33.3	0.589^1^
							
3^rd^ trimester	112	41.8	32	71.1	12	44.4	0.001^2^
							0.614^3^
							0.267^4^
**Restless legs syndrome (RLS)**							
1^st^ trimester	42	15.7	6	13.3	8	29.6	0.147^1^
3^rd^ trimester	76	28.4	9	20.0	13	48.1	0.483^2^
							0.077^3^
							0.029^4^

^1^One-way analysis of variance, ANOVA.

^2^Post hoc analysis (Tukey): non snorers vs. gestational snorers.

^3^Post hoc analysis (Tukey): non snorers vs. habitual snorers.

^4^Post hoc analysis (Tukey): gestational snorers vs. habitual snorers.

### Sleepiness

The mean Epworth Sleepiness Score for the entire study population increased from 7.9 to 8.7 from 1^st^ to 3^rd^ trimester (p < 0.001). The mean Epworth Sleepiness Score differed significantly between the three groups (Table 2). Post hoc analyses showed that the “gestational snorers” had a significantly higher mean Epworth Sleepiness Score than the “non snorers” in both the 1^st^ and the 3^rd^ trimester of pregnancy (p = 0.009 and p = 0.001), but this did not apply for the “habitual snorers” (p = 0.26 and p = 0.39). The prevalence of excessive daytime sleepiness (EDS) defined as Epworth Sleepiness score ≥10, only differed between the groups in the 1^st^ trimester of pregnancy, with significant difference proven between the “gestational snorers” and the “non snorers” (p = 0.001).

The “habitual snorers” had higher prevalence of restless legs syndrome than “gestational snorers” (p = 0.029) but not than “non snorers” in the 3^rd^ trimester of pregnancy.

### Obstetric outcome

There was a greater proportion of women in the snoring groups who developed preeclampsia or gestational hypertension (Table 3), but the affected women were too few to show statistical significance (p = 0.775, not shown in table). Of all the women in the study, nine (2.9%) developed preeclampsia during pregnancy and one woman was diagnosed with pregnancy-induced hypertension without leakage of protein to the urine. Out of nine women with preeclampsia; eight women were expecting their first child, and one had a history of preeclampsia in her previous pregnancy. Two of the women with preeclampsia also developed eclampsia. The recorded blood pressures during pregnancy did not differ between the groups (Table 2). Only one woman in the study developed gestational diabetes mellitus, she was categorized as “non snorer”.

**Table 3:** **Pregnancy outcome** (**n** = **339**)*

	**Non snorers**		**Gestational snorers**		**Habitual snorers**		
	(n = 267)*		**(n = 45)**		**(n = 27)**		
	**n**	**%**	**n**	**%**	**n**	**%**	**p value**
**Gestational week at delivery (mean/SD)**	39.5/1.4		39.5/1.5		39.7/1.5		0.668^1^
**Preeclampsia or gestational hypertension**	7	2.6	2	4.4	1	3.7	
**Normal vaginal delivery**	222	83.2	32	71.1	19	70.4	0.064^1^
**Vacuum extraction**	16	6.0	4	8.9	3	11.1	
**Acute caesarean section**	10	3.7	4	8.9	3	11.1	
**Elective caesarean section**	19	7.1	5	11.1	1	3.7	
**Bleeding >1000 ml**	11	4.1	4	8.9	2	7.4	
**Premature delivery (<37 weeks)**	6	2.2	2	4.4	1	3.7	
**Newborn Small for Gestational Age**	3	1.1	2	4.4	0		
**Newborn Apgar score <7 at 5 minutes age**	2	0.7	0		0		
**Fetal gender (n = 342)**							
**Boy**	134	50.0	25	55.6	8	27.6	0.768^2^
**Girl**	134	50.0	20	44.4	21	72.4	0.056^3^
							0.049^4^

*missing data about one women who moved to another city just before delivery.

^1^One-way analysis of variance, ANOVA.

^2^Post hoc analysis (Tukey): non snorers vs gestational snorers.

^3^Post hoc analysis (Tukey): non snorers vs habitual snorers.

^4^Post hoc analysis (Tukey): gestational snorers vs habitual snorers.

The mode of delivery did not differ between the groups (Table 3). The frequencies of larger bleeding, preterm delivery and adverse fetal outcome were too low to show statistical significance. The “habitual snorers” seemed to give birth to baby girls more often than the “non snorers” (p = 0.049).

## Discussion

In this prospective study the frequency of snoring more than doubled from the 1^st^ to the 3^rd^ trimester of pregnancy. The prevalence of snoring in the study group was 21.2% in the 3^rd^ trimester, which is slightly higher than reported in earlier prospective studies [4-6]. We chose to define the contributing women as “snorers” only when they stated that they snored often or always, since this is the standard definition used in sleep research. However, if all women who had never snored earlier but started to snore “sometimes” during pregnancy had been considered as a “gestational snorers”, the prevalence would have been even higher.

The most probable cause of snoring during pregnancy is a narrowing of the upper airways due to edema. Izci has reported smaller upper airway dimensions among pregnant women compared to non-pregnant controls [9]. High estradiol levels are the main cause of edemas in the airway mucosa [29] and other tissues. We investigated subjective peripheral edema of the legs, feet and hands and found that women who snored had a significantly higher degree of edema in the 3^rd^ trimester of pregnancy compared to the pregnant women who did not snore. This is novel knowledge. Weight gain might be another conceivable mechanism causing increased prevalence of snoring during pregnancy; this has also been reported in a previous survey [11]. We found no such association; instead our data imply that the BMI before pregnancy is of greater importance which is supported by data from a larger study [10]. This corresponds with risk factors for OSA in a non-pregnant, normal population [30,31] and the relationship between high BMI and snoring during pregnancy has also been reported in a recent cross-sectional survey [32]. High pre-pregnancy BMI has also been identified as a greater risk factor than weight gain for pregnancy and delivery complications such as antepartum stillbirth, cesarean delivery, instrumental delivery, preeclampsia and shoulder dystocia [33]. Smoking is a well-known risk factor for snoring in a general population [15], but has not been considered in this survey since the prevalence of smokers is very low (7%) among Swedish pregnant women [25].

We found that the women who started to snore, but not the habitual snorers, had higher Epworth Sleepiness Score and higher incidence of excessive daytime sleepiness than the non-snoring women already in early pregnancy, indicating that these women might already have had disturbed sleep before they started to snore. Association between sleepiness and snoring among pregnant women has, to the best of our knowledge, not been described earlier.

The relationship between snoring and restless legs syndrome found in this study has been analyzed in an earlier report [34] and has also been described in a general population [35].

As previously mentioned, there are numerous reports describing an association between sleep disturbances and adverse obstetric and neonatal outcome. One of the first was Franklin et al. who in 2000 published a retrospective study of 502 women where habitual snoring was identified as an independent risk factor for hypertension in pregnancy and growth retardation of the fetus, and correlated to preeclampsia and low Apgar score [13]. A recently published large American cohort study also found that new-onset snoring during pregnancy is a strong risk factor for gestational hypertension and preeclampsia [10]. There are theoretical possibilities that snoring might cause gestational hypertension and preeclampsia, since snoring is the cardinal symptom of obstructive sleep apnea (OSA), which in turn is known as an evident risk factor for the development of hypertension in the general population [36]. We found no significant association between snoring and preeclampsia.

In our study population, the “habitual snorers” more often gave birth to girls. Although there are reports describing that the sex of the expected child might impact the pregnancy in terms of plasma levels of human chorionic gonadotropin (hCG) [37] and pregnancy outcome [38], we do not consider this finding being of clinical relevance.

The main limitations of this study are connected to the subjective nature of many of the measured variables. No data about snoring was objectively recorded, but taken from the questionnaires, filled in by the pregnant women themselves. However, the majority of the women in the study were married and most of them were accompanied by their husband to the visits to the ACC, enabling, to some extent, verification of the snoring. Information about edema was also collected from the questionnaires, but the pregnant women are mostly very aware of the classification of edema since it is checked by the midwife at every visit to the ACC. Likewise sleepiness was reported by the women themselves, but the used instrument, the Epworth Sleepiness Score (ESS), is validated for measuring daytime sleepiness due to snoring, obstructive sleep apnea and other causes [39]. It has recently also been validated for measurement of sleepiness among pregnant women [40].

The drop-out rate from inclusion to the measurement in the 3^rd^ was 68%, however, drop-out analyses showed no difference between the non-responders in the 3^rd^ trimester and the remaining women.

Since most adverse obstetric outcome are rare, a large group of women are needed to verify statically assured correlations. The number of women included in this study was chosen on basis on an earlier study in this field [13], but post hoc power analysis show that almost the double sample size is needed for achieving a statistical power of 80% when investigating e.g. preeclampsia, that affects only about 3-4% of all pregnant women [41].

The major strength of the study is its prospective design. The women were recruited in early pregnancy and followed throughout their pregnancy and delivery in order to avoid recall bias. The women were consecutively collected from a normal pregnant population.

Since no objective data on snoring or OSA were collected, an obvious design element to be included in any future large prospective study would be a full respiratory recording on all contributing women.

## Conclusion

The frequency of snoring does increase during pregnancy and is related to high BMI at the start of pregnancy and higher frequency of edema in late pregnancy, but not to the degree of weight gain. Women who started to snore during pregnancy, but not the women who snored already from early pregnancy, had higher degree of sleepiness than the non snorers in both early and late pregnancy.

## Abbreviations

ACC: Antenatal care clinic; ANOVA: One-way analysis of variance; BMI: Body mass index; EDS: Excessive daytime sleepiness; ESS: Epworth sleepiness scale; ICD-10: International statistical classification of diseases and related health problems. 10th revision; OSA: Obstructive sleep apnea; RLS: Restless legs syndrome.

## Competing interests

The authors declare that they have no competing interests.

## Authors’ contribution

MS contributed to the data collection, data analysis, interpretation of data and the majority of the manuscript writing. ES contributed to the project design, interpretation of data and manuscript editing. ABW contributed to the data analysis and critical revision of the manuscript. AJ contributed to the project design, interpretation of data and manuscript editing. All authors have approved the final version of the manuscript to be published.

## Pre-publication history

The pre-publication history for this paper can be accessed here:

http://www.biomedcentral.com/1471-2393/14/15/prepub
